# Etiology and management of urinary retention in women

**DOI:** 10.4103/0970-1591.65396

**Published:** 2010

**Authors:** Amit Mevcha, Marcus J. Drake

**Affiliations:** Bristol Urological Institute, Southmead Hospital, Bristol, BS10 5NB, UK

**Keywords:** Bladder outlet obstruction, Fowler’s syndrome, urinary retention

## Abstract

Urinary retention (UR) can be defined as inability to achieve complete bladder emptying by voluntary micturition, and categorized as acute UR, chronic UR or incomplete bladder emptying. UR is common in elderly men but symptomatic UR is unusual in women. The epidemiology of female UR is not well documented. There are numerous causes now recognized in women, broadly categorized as infective, pharmacological, neurological, anatomical, myopathic and functional; labeling symptoms as having a “psychogenic basis” should be avoided. Detrusor failure is often an underlying factor that complicates interpretation. Initial management includes bladder drainage (intermittent or indwelling catheterization) if the woman is symptomatic or at risk of complications, and correcting likely causes. Investigations should be focused on identifying the underlying etiology and any reversible factor. A detailed history, general and pelvic examination are needed; urine dipstick analysis, routine microscopy and culture, and pelvic and renal ultrasound are suitable baseline investigations. Urodynamic tests are required in specific situations. Urethral dilatation has a limited role, but it should be considered if there is urethral stenosis. Definitive management requires correction of cause where possible and symptom management where no correctable cause is detected. Follow-up is needed for monitoring response to treatment, detection of complications and symptom control. Fowler’s syndrome is a specific group diagnosed on urethral sphincter electromyogram, representing a very challenging clinical scenario.

## INTRODUCTION

Physiologically, voiding requires a coordinated (synergic) bladder contraction and outlet relaxation, with sustained detrusor contraction achieving complete bladder emptying. Clinical problems can give rise to voiding lower urinary tract symptoms (LUTS) including poor urinary stream, intermittent flow, terminal dribbling and post-micturition dribbling.[1] Urinary retention (UR) is a severe impairment of voiding, which can be defined as inability to achieve complete bladder emptying by voluntary micturition. Acute urinary retention (AUR) is a painful bladder distension, which usually presents as an emergency. Chronic urinary retention (CUR) is a non-painful bladder distension, leading to overflow dribbling and risk of impaired upper urinary tract function. Incomplete bladder emptying signifies the presence of a post void residual (PVR). Agreement as to what constitutes a clinically significant PVR has not been achieved, with absolute volumes, proportion of bladder capacity, or presence of relevant symptoms all included in some contexts. Pathophysiologically, UR is a consequence of one or more of: reduced bladder contractility, poorly sustained detrusor contraction, lack of an adequate anatomical outlet, deficient outlet relaxation, or impaired neurological coordination of the voiding process.

For male patients, particularly in older age groups, bladder outlet obstruction (BOO) is comparatively common, usually as a consequence of benign prostate enlargement (BPE). Diagnostic criteria for male BOO have been agreed based on the relationship between the detrusor pressure at maximum flow (P_det_ Q_max_ ) and the concurrent maximum flow rate (Q_max_ ) to derive the BOO index (BOOI).[2] The same parameters can also be used to calculate a contractility index (BCI). Prior to prostate surgery, the BOOI and the BCI help predict likely benefits resulting from operation. Furthermore, the epidemiology of voiding LUTS in BPE has been studied to some extent. For example, acute urinary retention (AUR) occurred in 7% of men with BPE over a four-year period.[3]

For women, UR is a contrasting situation, resulting from a much more diverse set of conditions. Accordingly, epidemiological research is difficult and the natural history of the various underlying conditions is only minimally understood. The incidence of UR in women is not well documented. One Scandinavian study revealed an incidence of AUR in women of 7 per 100,000 population per year; the male to female ratio was 13:1.[4] In general, female UR is more frequently described in small case series or case reports with unusual causes. This diversity and lack of consensus on management means that treatment outcomes are harder to predict than in the male.

In this review article we summarize what has been published on the subject relating to the anatomical basis, assessment and management of the female BOO, with particular emphasis on iatrogenic obstruction, postoperative urinary retention and Fowler’s syndrome.

## DEFINING URINARY OBSTRUCTION

Although there are no known universally accepted or standardized criteria for BOO in women, several useful studies have examined the question. Diokno and colleagues defined BOO in women in 1984 on the basis of videourodynamic studies.[5] They defined BOO when the detrusor pressure was ≥ 60 cm of water and the peak urine flow rate was less than 15 ml/sec, with relaxation of the external sphincter, and without funnelling of the bladder neck during voiding. The diagnosis of BOO was established in three of a large pool of patients referred with voiding symptoms, an elevated post void residual, or performing self-catheterization.

Nitti and colleagues derived criteria from evaluation of the videourodynamic studies of 261 women for non-neurogenic voiding function and defined BOO as radiographic evidence of obstruction between the bladder neck and the distal urethra in the presence of a sustained detrusor contraction of any magnitude, usually associated with reduced flow rate or delayed flow.[6]

Blaivas and Groutz derived a nomogram from studying 50 women with somewhat diverse etiologies, who were concluded to be obstructed on clinical grounds.[7] Their study defined BOO by presence of free Q_max_ ≤ 12 ml/sec in repeated free flow studies, combined with a sustained detrusor contraction and P_det_ Q_max_ ≥ 20 cm H_2_O in a pressure flow study, or presence of obvious radiographic evidence of BOO with a sustained detrusor contraction of at least 20 cm of water and poor Q_max_ - regardless of free Q_max_ or inability of void with the transurethral catheter in place despite a sustained detrusor contraction as above. Their nomograms plotted parameters from two separate voids – the maximum flow rate from a free (uncatheterized) flow on the X-axis and the detrusor pressure at maximum flow measured during voiding cystometry on the Y-axis. Whilst use of two separate voids is counter-intuitive, the presence of a urodynamic catheter in the urethral lumen implicitly must alter the gauge of the outlet available for urine flow, potentially confounding the interpretation of the outlet gauge. This nomogram enables differentiation not only between obstructed and non-obstructed patients but also between various degrees (mild, moderate and severe) of BOO.

Chassange and colleagues derived the relationship between Q_max_ and P_det_ Q_max_, comparing women with anatomical outlet obstruction versus women with stress urinary incontinence.[8] It was concluded that Q_max_ 15 ml/s or less and P_det_ Q_max_ 20 cm H^2^ O or more are reasonable pressure-flow parameters to define BOO in women (giving sensitivity, specificity, positive predicted value and negative predictive value as 74.3, 91.1, 70.3 and 92.6 respectively). These criteria were revised in subsequent publications.[9,10] De Freitas et al. concluded that the combination of the maximum flow rate of up to 12 ml/s with a detrusor pressure in excess of 25 cmH_2_ O represented cutoff parameters with the highest sensitivity and specificity for BOO in women.[9]

The various approaches to diagnosing BOO in women were compared by Akikwala and colleagues, studying 91 patients, in whom obstruction was suspected clinically in 25 women.[11] All the patients were classified as obstructed or unobstructed by the different criteria alluded to above. They calculated that BOO was diagnosed with at least one diagnostic approach in 40 of the 91 study population; nine were obstructed on all the criteria, while a different nine fulfilled only one criterion. The Blaivas and Groutz nomogram appeared to diagnose more women as being obstructed. In the opinion of the authors, the revised criteria[9] appeared to underestimate the prevalence of BOO, concluding that the best concordance was between the 1998 criteria[8] and videourodynamic observation.[5]

A key issue in diagnosing BOO is the difficulty gauging the contractility of the bladder during voiding. Impairment of bladder contractility appears to be a feature of the ageing bladder[12] and can be expected to affect urinary flow adversely, potentially leading to over-diagnosis of outlet obstruction. A stop-test, in which the urinary stream is interrupted during voiding, should result in a significant rise in isovolumetric detrusor pressure during the flow interruption (p_det.iso_ ).[13] Where the rise in pressure is small, the contractility can be presumed to be reduced, but universally-agreed criteria are lacking. Stop-tests are undertaken in some centers, but the inhibitory effect on the voiding reflex and the likely need for a second filling and voiding study, means their use is not widespread. An impression of the bladder contractility can be inferred by simple examination of the detrusor pressure during flow. A fluctuating low-pressure detrusor contraction, corresponding with a fluctuating flow, particularly if the patient supplements the bladder contraction by abdominal straining, does suggest reduced bladder contractility. In these patients, caution is needed with interpretation. While low flow with high detrusor pressure may signify BOO, low flow with low detrusor pressure does not exclude BOO. Ascertaining whether a raised PVR is a consequence of BOO or reduced contractility is key dilemma for the managing clinician.

## CAUSES AND MANAGEMENT OF FEMALE BLADDER OUTLET OBSTRUCTION

The Table 1 lists causes of female BOO and UR. The basis of obstruction is subdivided into urethral compression, bladder neck distortion or luminal occlusion.[14] To these can be added functional issues of non-relaxing sphincter dysfunction and impaired neurological coordination of the outlet components of the lower urinary tract, compounded by reduced bladder contractility. Where all other factors have been excluded, UR might be labeled “psychogenic”,[15] but this diagnosis should be applied following comprehensive evaluation and with considerable circumspection. Case reports have described various scenarios, such as benign inflammatory nervous disease[16] (sacral herpes, meningitis), uterine leiomyoma,[17] cytomegalovirus cystitis,[18] eosinophilic cystitis[19] and incarcerated gravid retroverted uterus.[20]

**Table 1:** Causes of urinary retention and bladder outlet obstruction

Anatomical	
Extrinsic	Pelvic organ prolapse
	Gynecological e.g. uterine fi broid, tumor
	Poorly fitting pessary
	Post anti-incontinence procedure
Urethral	Stricture
	Meatal stenosis
	Thrombosed urethral caruncle
	Diverticulum
	Skene’s gland cyst or abscess
Luminal	Stone
	Bladder/ urethral tumor
	Ureterocoele
	Foreign body
Impaired detrusor contractility	Senile bladder change
	Diabetes mellitus
	Neurological disease (lower motor neurone lesions)
Functional	
Impaired coordination	Primary bladder neck obstruction
	Fowler’s syndrome
	Pseudo-dyssynergia
	Detrusor-external sphincter dyssynergia
	Neurological disease (upper motor neurone lesions)
Peri-operative	Pain
	Analgesia or anesthetic e.g. epidural
Infective/inflammatory	UTI
	Acute vulvovaginitis
	Vaginal lichen planus/sclerosis
	Genital herpes
Pharmacological	Opiates
	Antipsychotics
	Antidepressants
	Antimuscarinics
	α-adrenergic agonist

Detailed history, abdominal, pelvic and neurological examination should be carried out. Immediate management of AUR requires bladder decompression with catheterization, either indwelling or intermittent catheterization (IC). IC not only avoids potential morbidity with an indwelling catheter but also allows monitoring of return of voiding function, which is usually presaged by a reduction in PVR.[21,22] Any urinary tract infection should be treated with appropriate antimicrobial therapy. Other reversible causes such as prolapse should be identified and rectified. Further radiological and pressure-flow investigation may be required in some patients. Pelvic ultrasound occasionally reveals relevant findings that may contribute to the symptoms in women with urinary retention. Gynecological causes are unusual, such as large uterine fibroids, but they must be considered in undertaking complete evaluation.

The utility of flexible cystoscopy under local anesthesia is debatable, but in a minority of cases it can provide additional anatomical information, for example, presence of intraluminal foreign body. Many women undergo urethral dilatation as a part of management, though there is no evidence to support it.[23,24] However, in patients with primary bladder neck obstruction, reported in 9-16% of women with BOO, bladder neck incision (BNI) or transurethral resection can improve voiding. BNI can be performed in the midline or at the 5 and 7 o’clock positions, perhaps combined with resection of intermediate tissue. Various efficacy results have been published from small series and there is a recognized risk of causing subsequent stress urinary incontinence (SUI).[25–27] In women, α-blockers can lower the resting urethral pressure.[28] However, they achieve no significant difference in success of trial without catheter compared against placebo.[29]

## POSTOPERATIVE URINARY RETENTION

UR is a poorly-understood yet well-recognized complication postoperatively in both men and women. Contributing factors include traumatic instrumentation, bladder over-distention, reduced contractility of bladder, increased outlet resistance, nociceptive inhibitory effect, pharmaceutical influences, preexisting outlet pathology and decreased micturition reflex activity. Various studies have shown that specific types of anesthesia and analgesia can increase the risk of postoperative urinary retention. In a review of more than 3000 obstetric deliveries Olofsson and colleagues demonstrated that patients who received epidural anesthesia had an increased risk of UR,[30] in fact, urinary retention post partum is probably underdiagnosed in general.[31] Another study compared regional anesthesia (spinal or combined spinal and epidural) and non-regional anesthesia (general, monitored anesthesia with sedation, and local) and incidence of UR following outpatient mid-urethral sling procedure, concluding that regional anesthesia is associated with higher risk of acute retention.[32] Gallo et al., looked at the effect of low-dose naloxone in patients who received morphine as patient-controlled analgesia following orthopedic surgery and showed that fewer patients required catheterization postoperatively.[33]

## IATROGENIC BOO RELATED TO SURGERY FOR TREATMENT OF URINARY INCONTINENCE

### Stress urinary incontinence surgery

Many women have partial UR in the early stages after SUI surgery. Presumably, several factors will be involved, such as the anesthetic agents employed; local discomfort or the analgesic agents used to treat the discomfort; edema or hematoma formation; altered voiding dynamics as a consequence of outlet realignment. Voiding usually recovers comparatively swiftly, but close surveillance is needed to check resolution of symptoms and PVR, and ensure that progression to full AUR and emergency issues do not ensue.

Full AUR is rare postoperatively, but needs intensive attention at the outset to ensure bladder overdistension injury does not occur. Timing of trial without catheter has to be individualized, dependent on resolution of identifiable remediable factors. If spontaneous voiding does not result, an early decision needs to be taken on management strategy. Delaying intervention beyond one month may result in a permanent impairment a-s a consequence of fibrotic in-growth into the tape interstices. Factors in the history comprise whether urinary retention is complete or partial and whether it is improving with time. Key features of past medical history should be re-evaluated. For example, any current or previous neurological problem can alter the neural control of the urinary tract, leading to temporary or permanent difficulty with voiding, unrelated to tape placement. It is also valuable to review the preoperative urodynamics to re-evaluate voiding function, and to review technical problems during the operative placement or subsequent complications, such as hematoma. Physica-l examination is crucial; for some women with over-tight mid-urethral sling placement it is possible to feel the mid-urethral compression during vaginal examination. In these patients tape incision has a realistic chance of restoring voiding. Less commonly, it is possible to feel the anterior vaginal wall drawn up behind the pubis, suggesting that incision of the tape alone is unlikely to restore voiding and that additional per-operative attention to address the abdominal retropubic component of the tape will be needed to re-establish voiding. Finally, when physical examination reveals that the anterior vaginal wall lies in an entirely undisturbed location, it can be anticipated that impaired voiding is not a consequence of the urethral compression by the tape; tape incision or excision is then unlikely to achieve re-establishment of voiding. In this last group it is appropriate to consider proceeding to formal voiding cystometry.

Various publications have now described the outcomes of releasing mid-urethral tapes surgically, including the restoration of voiding and the recurrence of incontinence as a consequence. If proceeding to cut a mid-urethral tape, cystoscopy should be undertaken at the same time to ensure that no other potential tape-related complications have also arisen. Patients have to be evaluated on an individual basis, rather than simply proceeding to tape incision based purely on failure to achieve normal voiding within a certain time frame. Nonetheless, it is important to proceed in a timely manner if the assessment does suggest that the tape is causing the obstruction, since ongoing mesh fibrosis, potentially compounded by decompensation of detrusor function, may lead to permanent impairment if avoidable delay intervenes.[34] Hong et al. described 32 patients who had voiding dysfunction, of 375 undergoing a transvaginal tape (TVT) placement.[35] Eighty-eight per cent needed intermittent self-catheterization which was undertaken for less than one month. The median time to normal voiding was nine days. Four had the tape sectioned at 61 days, in whom three had recurrence of SUI. Return of incontinence following tape sectioning is seen in a substantial proportion;[36] obstructive symptoms tend to improve, whilst storage symptoms tend to remain unchanged.

Other forms of SUI have also been associated with BOO. Colposuspension can lead to urethral compression or distortion,[37] particularly where sutures are placed close to the urethra. The Marshall-Marchetti-Krantz procedure appeared to carry a definite risk of causing urethral distortion and obstruction in a proportion of patients.[38] Bone anchor slings also led to some obstructive complications.[39] In some cases the placement of the tape to cause urethral compression can be a deliberate therapeutic approach. For example, a compressing autologous sling can be an intended therapeutic strategy used in the management of intrinsic sphincter deficiency, for those women trained in IC preoperatively. A similar approach using TVT should not be advocated, in view of the potential risk of late urethral erosion with over-tight tapes, which can occur many years after surgery.[40]

### Urgency urinary incontinence surgery

Some forms of surgical management of refractory urgency urinary incontinence (UUI) or overactive bladder (OAB) aim to enhance urinary reservoir function by reducing bladder contractions during the storage phase. This can also impair bladder contraction needed for voiding. Thus, patients undergoing successful augmentation cystoplasty or detrusor myectomy will have a substantial PVR in a high proportion of cases.[41,42] Intravesical botulinum toxin injections, an unlicensed procedure for management of OAB,[43] also carry a significant risk of UR, which appears to increase with repetition of injections following return of symptoms.[44] Risk factors for UR and regimes achieving optimal balance of sustained efficacy with minimized adverse effects are still uncertain; patients should be aware of the risk accordingly, and should be willing to accept the possibility of needing IC subsequently.

## FOWLER’S SYNDROME

Fowler’s Syndrome affects young women after the menarche, who develop painless retention at high bladder volumes, often following apparently unconnected precipitating events, such as minor surgery.[45] Often, history of prior LUTS is minimal and most of the women will not report any prior urinary tract problems. It is estimated that around 40% of women affected have Polycystic Ovary Syndrome. It is important to exclude occult or undiagnosed neurological problems as a cause. The scientific explanation for the underlying sphincter problem in Fowler’s syndrome is not understood. It has been hypothesized that changes in the ion channels of the skeletal muscles of the urinary sphincter may be affected by the hormonal environment of the menarche (“hormonal channelopathy”) leading to abnormal communication directly between muscle cells (ephaptic transmission). As a consequence, the sphincter becomes overactive and hypertrophic, and reacts excessively to direct stimulation.

Diagnostic criteria include: UR of at least 1 liter on at least one occasion; exclusion of other causative factors; raised maximum urethral closure pressure on urethral pressure profilometry; increased sphincter volume on ultrasound or MRI assessment; and; a characteristic urethral sphincter EMG. Difficulties with IC can be profound—insertion of the catheter can be straightforward but then discomfort may develop, as if the sphincter were gripping the catheter, leading to consequent difficulty on catheter withdrawal. Flow rate patterns tend to be interrupted. Small volumes often are passed by micturition, leaving substantial PVR.

The most specific diagnostic test for Fowler’s Syndrome is a urethral sphincter EMG (USEMG), which differs from the pelvic floor EMG generally used for neurourological patients. In USEMG, the EMG needle is placed to one side of midline in the anterior vaginal wall, at the mid-urethral point, and advanced on to the dorsal aspect of the urethra. The neurophysiologist undertaking the test has to pay special attention to the audio signal being generated by the EMG, which confirms successful entry into the sphincter zone. The diagnostic parameter for Fowler’s Syndrome is an audio signal likened to the sound of whale noises in the ocean. Pelvic floor EMG often is non-diagnostic in this patient group.

Management of Fowler’s syndrome is specialized, and a sympathetic approach and consideration of psychological elements are essential. Strong efforts should be made to reduce the polypharmacy that many of these patients have, particularly attempting to discourage use of opiate drugs. For those patients manifesting the characteristic EMG signal who are unable to tolerate IC, the treatment of choice is sacral nerve stimulation (SNS),[46] which can achieve normal voiding in a significant proportion of women affected. Management is difficult in patients without the characteristic EMG signal, or those patients where the SNS percutaneous needle electrode test fails to elicit a significant improvement in symptoms. Suprapubic catheter placement is generally unsatisfactory in younger women. No drug treatment has yet been established as deriving any substantive benefit. Botulinum injection into the urethral sphincter has not been tested on a systematic randomized basis. Ultimately, reconstructive surgery using a continent diversion (Mitrofanoff procedure) may be necessary.

## CONCLUSION

Bladder outlet obstruction is uncommon in women. Careful evaluation including detailed history, physical examination and urodynamics is required to find out any reversible pathology; formal urodynamic criteria have been proposed, but no standardized approach has been achieved. IC may be necessary for the management of BOO. Surgical treatment in women should be approached with caution as it carries significant risk of injury to sphincter and incontinence, and intervention requires an individualized approach based on careful evaluation. Fowler’s syndrome is a specific group representing a very challenging clinical scenario.
